# Genetic Variability Impacts Genotoxic and Transcriptome Responses in the Human Colon after the Consumption of Processed Red Meat Products and Those with Added Phytochemical Extracts

**DOI:** 10.3390/nu16030425

**Published:** 2024-01-31

**Authors:** Julia N. DeBenedictis, Esther Baars, Juan Ochoteco-Asensio, Simone G. van Breda, Theo M. de Kok

**Affiliations:** Toxicogenomics Department, GROW School of Oncology & Reproduction, Faculty of Health, Medicine & Life Sciences, Maastricht University, 6211 LK Maastricht, The Netherlandsjuan.ochoteco@irta.cat (J.O.-A.);

**Keywords:** phytochemicals, colorectal cancer prevention, nutrigenomics, personalized nutrition, genetic variability

## Abstract

The PHYTOME study investigated the effect of consuming processed meat products on outcomes related to colorectal cancer risk without testing the impact of genetic variability on these responses. This research aims to elucidate the genetic impact on apparent total N-nitroso compound (ATNC) excretion, colonic DNA adduct formation, ex vivo-induced DNA damage, and gene expression changes in colon biopsies of healthy participants. Through a systematic literature review, candidate polymorphisms were selected and then detected using TaqMan and PCR analysis. The effect of genotype on study outcomes was determined via a linear mixed model and analysis of variance. Machine learning was used to evaluate relative allele importance concerning genotoxic responses, which established a ranking of the most protective alleles and a combination of genotypes (gene scores). Participants were grouped by GSTM1 genotype and differentially expressed genes (DEGs), and overrepresented biological pathways were compared between groups. Stratifying participants by ten relevant genes revealed significant variations in outcome responses. After consumption of processed red meat, variations in NQO1 and COMT impacted responses in ATNC levels (µmol/L) (+9.56 for wildtype vs. heterozygous) and DNA adduct levels (pg/µg DNA) (+1.26 for variant vs. wildtype and +0.43 for variant vs. heterozygous), respectively. After phytochemicals were added to the meat, GSTM1 variation impacted changes in DNA adduct levels (−6.12 for deletion vs. wildtype). The gene scores correlated with these responses and DEGs were identified by GSTM1 genotype. The altered pathways specific to the GSTM1 wildtype group included ‘metabolism’, ‘cell cycle’, ‘vitamin D receptor’, and ‘metabolism of water-soluble vitamins and co-factors’. Genotype impacted both the potential genotoxicity of processed red meat and the efficacy of protective phytochemical extracts.

## 1. Introduction

In addition to age and family history, environmental factors like diet significantly contribute to the risk of developing colorectal cancer [1,2]. Notably, processed red meat is classified as a Group 1 human carcinogen by The International Agency for Research on Cancer (IARC) following a comprehensive analysis of 800 studies [3,4]. Research findings indicate that consuming 50 g per day of processed meat increases the risk of colorectal cancer by 18% [3]. One of the proposed mechanisms by which processed red meat exerts its carcinogenic effect involves the nitrites they contain. Nitrites are critical to the formation of endogenous N-nitroso compounds (NOCs), which form a versatile and potent class of potential carcinogens. Animal studies have provided strong evidence of the carcinogenic potential of NOCs, and some epidemiological studies also suggest a correlation between NOC exposure and cancer risk in humans [5,6,7]. These compounds, along with their precursors, are present in meat in the form of amines and amides. In the case of processed meats, nitrites and nitrates are also present and act as precursors giving rise to endogenous NOC formation in the colon. The heme found in red meat further catalyzes the formation of NOCs. One of the ways NOCs elevate the risk of colorectal cancer is by generating covalently bound DNA adducts, which may result in DNA breaks or mutations that could initiate the development of cancerous cells [8]. Notably, a characteristic pro-mutagenic DNA adduct formed by NOCs, O6-Carboxymethyl Guanine (O6-MeG), has been shown to increase significantly with a high red meat diet [9,10]. NOCs have been shown to exert genotoxic and mutagenic effects by impacting multiple cellular processes, including alterations in DNA damage repair, cell cycle regulation, and apoptosis pathways, all of which can contribute to the onset of colorectal carcinogenesis [11,12,13,14].

Despite the role of nitrite in the formation of NOCs, meat manufacturers still add nitrite to their products. This is done to extend the shelf life of meat products and to ensure that they have an appealing red color [15,16,17]. Processed red meat consumption remains high in North America and Europe [18,19]. Average intakes exceed the recommended upper limits by the UK Scientific Advisory Committee on Nutrition of 70 g per day, or the “very little, if any” recommendation by the World Cancer Research Fund, despite its known cancer-causing potential [19].

Conversely, the phytochemicals present in fruits and vegetables have antimicrobial, antioxidant, and anticarcinogenic properties, and are known to play a role in reducing colorectal cancer risk [20]. Their antimicrobial properties make them a suitable substitute for added nitrite in meat products, extending their shelf life. In the context of colorectal cancer, these compounds inhibit the formation of NOCs, acting at the level of carcinogenic compound kinetics and cellular protection in the colon [21].

**Figure 1:**
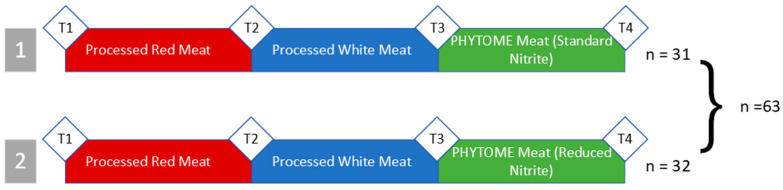
PHYTOME Study Design. T = test day. Group 1 was randomized to consume standard-nitrite PHYTOME meat, and group 2 was randomized to receive reduced-nitrite PHYTOME meat. Each intervention period lasted 14 days.

The PHYTOME study is a parallel human nutrition intervention study that aimed to examine the effect of different meat products on DNA damage, NOC excretion, and gene expression in the colons of healthy volunteers (Table A1) [22]. In particular, it aimed to test if adding phytochemical-rich extracts to processed red meat (with standard nitrite or reduced nitrite levels) reduced its carcinogenic potential as measured by these outcomes. Healthy subjects consumed 300 g per day of three different types of meat for two weeks each. The first intervention was standard processed red meat (T2), followed by white meat (T3), and then finally processed red meat enhanced with phytochemical-rich natural extracts and standard or reduced levels of nitrite (T4) (Figure 1).

To determine the meat dosing, we relied on prior research and data from the national food consumption survey in the Netherlands, which indicated an average daily meat intake of 150 g per day among the population [23]. Consequently, we implemented a personalized meat dosage based on body weight, amounting to 3.75 g per kilogram body weight, with an upper limit of 300 g per day. This amount maximizes intake while still falling within the typical daily meat consumption range in the Netherlands [13,23,24].

The processed red meat package included a variety of traditional processed red meat products, such as cooked ham, raw ham, cooked sausage, dry sausage, and dry cured ham. These products adhered to conventional processing standards and contained typical nitrite levels. The white meat package featured unprocessed chicken and turkey with cooking instructions. The phytochemical-enriched red meat products contained the same products included in the first intervention, maintaining either standard nitrite levels (group 1) or reduced nitrite levels (group 2), while incorporating natural extracts (Table 1). Fish was excluded throughout the study due to its high amine content, which could potentially interfere with the analyses [25]. Throughout the intervention, participants maintained a daily intake of fruits and vegetables at a modest but acceptable level, consisting of 50 g of vegetables and one piece of fruit.

The phytochemical-enriched meat products are called PHYTOME meat. PHYTOME meat products were prepared at two different nitrite levels: standard nitrite (group 1) and reduced nitrite (group 2). In group 1, nitrite was added following standard manufacturing practices and European regulations [26], while in group 2, nitrite levels were reduced or eliminated while preserving the traditional sensory characteristics of the products. Both meat types were enriched with carefully selected combinations of natural antioxidants and bioactive compounds from plant extracts based on scientific evidence for their antioxidant, chemopreventive, and antimicrobial properties [22]. These extracts met various criteria, including natural origin, commercial availability, and compatibility with manufacturing processes. Different trial versions of innovative meat products were developed with these extracts, ensuring they did not adversely affect meat quality or sensory attributes. Commercial extracts from various plants, such as Polygonum cuspidatum, Sophora japonica, green tea, white grape, rosemary, oregano, sage, melissa, and acerola, were incorporated into meat mince or curing brines to provide polyphenols and ascorbic acid, known for their potential cancer risk reduction benefits. Manufacturing methods were adjusted according to the type of meat product to incorporate natural extracts, aiming to achieve a polyphenol content per serving reported to reduce cancer risk [27,28]. The concentrations of polyphenols and ascorbic acid in the final meat products therefore varied depending on the level of nitrite, added extracts, and processing techniques employed, but efforts were made to optimize bioactive compound levels while maintaining product quality and sensory attributes [29] (Table 1).

Concentrations of polyphenols in dry and cooked sausages were about 2–2.5 g/kg (as gallic acid equivalents) and 0.5 g/kg, respectively [29]. Dry-cured hams treated with brine vacuum impregnation [30] contained approximately 1–1.5 g/kg of polyphenols and 0.4 g/kg of ascorbic acid. Cooked and raw hams processed with brine injection had lower levels of polyphenols and ascorbic acid, both below 0.5 g/kg and 0.1 g/kg, respectively. To ensure safety when processing meats without nitrite or with nitrite levels below 50 mg/kg, we implemented an early cold drying treatment (0–3 °C) for dry sausages, dry sausage southern style, and dry-cured ham [31]. This effectively reduced water activity (aw) and decreased pH in a controlled manner [32,33]. In summary, PHYTOME meat products were carefully formulated to offer health benefits without compromising taste and quality.

Fecal excretion of NOCs, measured as Apparent Total N-nitroso Compound (ATNC) levels, significantly decreased after consumption of the PHYTOME meat as compared to the traditionally processed red meat. However, no effect was found on O6-MeG adduct levels in colonic DNA or DNA strand breaks (induced ex vivo in fecal water-exposed Caco-2 cells) for this comparison. ATNC levels were significantly higher after consuming standard red meat compared to white meat, and the ATNC levels after consuming white meat were significantly higher than the PHYTOME meat with reduced nitrite. The lowest DNA adduct levels were found after consumption of the white meat products. The adduct levels were significantly higher at baseline, after consumption of the processed red meat products, and after consumption of the PHYTOME meat products as compared to white meat. Similarly, DNA strand break levels were significantly lower in the white meat group compared to the standard red meat group. Transcriptomic analysis of colonic tissue microarray data revealed that changes in gene expression related to cell proliferation were the predominant molecular mechanisms affected by the addition of phytochemicals. However, the gene expression analysis performed on participant colon tissue did not result in statistically significant differentially expressed genes (DEGs) in colonic tissue after consuming the standard red meat compared to the PHYTOME meat. While the PHYTOME study yielded significant findings regarding NOC excretion after phytochemical-enriched meat consumption, subsequent decreases in O6-MeG adduct level, DNA strand breaks, and gene expression were not identifiable. A large inter-individual variation in responses was observed, which suggests the potential influence of genetic factors. Overlooking unmeasured gene–diet interactions could lead to a misinterpretation of the intervention as ineffective at certain levels when it may be effective for those of a particular genetic background.

Polymorphisms in genes coding for metabolizing enzymes can alter the metabolic response of an individual, resulting in a different effect of a dietary intervention [34]. These variations often result in two main categories: ‘slow’ and ‘fast’ metabolizers, representing the altered and often reduced efficiency of an enzyme from a mutation in the corresponding gene. This distinction has notable implications for the metabolism of phytochemicals and pre-carcinogens. In the case of a phytochemical requiring metabolic activation, slow metabolizers may experience a reduced biological effect due to diminished enzyme function. Slow metabolizers may also exhibit less efficient conversion of pre-carcinogenic compounds into their harmful forms, resulting in reduced harm after these exposures. Conversely, slower metabolizing enzymes may also result in reduced clearance of the bioactive forms of some phytochemicals, resulting in a sustained physiological impact, whereas a slow detoxification enzyme may lead to a harmful build-up of potentially genotoxic compounds [35].

Inter-individual genetic variation could therefore influence the formation of NOCs and the induction of DNA adducts and DNA strand breaks following the different interventions. Moreover, variability in genetic responses may have previously obscured the detection of gene expression changes. Therefore, this study aims to evaluate the effect of genetic variability on the excretion of NOCs, the formation of colonic DNA adducts, ex vivo-induced DNA damage, and accompanying gene expression changes after consumption of different meat interventions. To accomplish this, a systematic review was performed to determine the most relevant evidence-based polymorphisms to measure in this study population. Stored participant samples were genotyped and the outcomes of the PHYTOME study were stratified by genotype to evaluate the effect of genetic variation on responses to the intake of different processed meats. Also, a gene score was evaluated to determine the relative importance of each allele in contributing to the responses in outcomes.

## 2. Materials and Methods

### 2.1. Literature Search

A literature search was conducted via PubMed and Web of Science. PRISMA guidelines were used in this systematic review [36]. The population, intervention, comparison, outcome, and study (PICOS) model was used to develop inclusion criteria and search terms [37].

#### 2.1.1. Identification of Studies

PubMed and Web of Science were used to search articles published between January 2010 and May 2021, associating genetic variants related to meat or phytochemicals (intervention) and increased or decreased risk (comparison) of colorectal cancer or other outcomes of the PHYTOME study (outcome) in healthy individuals (population).

The following keywords were used: (“SNP” OR “polymorphism” OR “single nucleotide polymorphism” OR “variant”) AND (“colorectal cancer” OR “DNA damage” OR “nitroso compounds” OR “cell proliferation” OR “DNA adduct”) AND (“phytochemical” OR “fruit” OR “vegetables” OR “meat” OR “nitrite” OR “nitrate”).

#### 2.1.2. Eligibility Criteria

The inclusion criteria were: (a) publication date between January 2010 and May 2021; (b) written in English or Dutch; (c) published in a peer-reviewed journal; (d) in humans; (e) studied single nucleotide polymorphisms (SNPs), polymorphisms, or genetic variants; (f) outcomes include colorectal cancer risk or other outcomes of the PHYTOME study; (g) outcomes include formation of nitroso compounds, phytochemical action, and the metabolism of meat, nitrite, and nitrate.

Studies were excluded from the review if they were: (a) in animals or plants, (b) a repeated publication, (c) articles with only an abstract available, (d) unpublished theses or dissertation studies, (e) not published in a peer-reviewed journal.

#### 2.1.3. Study Selection and Data Collection

After the initial literature search was conducted, the title and abstract of each study were screened. Next, potentially relevant studies were further assessed for eligibility. The study selection process followed PRISMA guidelines.

#### 2.1.4. Polymorphism Selection Criteria

To select promising candidate genes, selection criteria were defined. At first, the candidate gene was required to have a direct relationship to colorectal cancer, to the metabolism of phytochemical action, or to outcomes of the PHYTOME study [22]. Secondly, the candidate gene should have a polymorphic variant. The candidate gene should not be synonymous and should alter the efficacy, activity, or specificity of the protein product. Finally, the prevalence of the polymorphism within the population should be at least 20%.

### 2.2. Practical Research

#### 2.2.1. Study Samples

DNA for genotyping had already been collected with the PHYTOME study. DNA originated from colon biopsies taken from the 63 participants in the PHYTOME study. DNA was isolated according to standard protocols [22]. For genotyping, DNA was diluted to reach a concentration of 10 ng/µL in TE buffer.

#### 2.2.2. Genotyping

Participants of the PHYTOME human dietary intervention study were genotyped for ten polymorphisms (CYP2E1 Rs28371744, CYP1A2 Rs35694136, NAT1 Rs4986783, NAT2 Rs1799931, NQO1 Rs1800566, XRCC1 Rs25487, COMT Rs4680, MGMT Rs16906252, and deletion of GSTM1 and GSTT1 polymorphisms). Genotyping for CYP2E1, MGMT, and the deletion of GSTM1 and GSTT1 polymorphisms was carried out using a modified multiplex PCR method. Genotyping for CYP1A2, NAT1, NAT2, NQO1, XRCC1, and COMT1 SNPs was carried out using TaqMan single nucleotide polymorphism genotyping assays (Thermo Fisher Scientific, Waltham, MA, USA).

#### 2.2.3. PCR Analysis

Multiplex PCR was used for the detection of GSTM1*0 (rs366631) and GSTT1*0 (rs17856199) polymorphisms. β-globin was used for the internal control for GSTM1*0 and GSTT1*0. For the detection of CYP2E1 (Rs28371744) and MGMT (Rs16906252), singleplex PCR was performed. The first cycle was performed for 3 min at 95 °C, and then 40 cycles of 60 s alternating from 95 °C, 56 °C, and 72 °C, followed by 10 min at 72 °C and ending at 20 °C. In Table 2, the amplified fragment sizes and primer sequences can be found for these genes.

Gel electrophoresis was used for the determination of genotype. The presence of a band in the 2% agarose gel indicated the wildtype genotype, while the absence indicated the null genotype of GSTM1 and GSTT1. The presence of a band at 729 bp indicated the wildtype variant for CYP2E1, whereas, without the insertion, a band could be found at 633 bp. To determine the genotype in the MGMT gene, digestion with Hhal (Thermo Fisher Scientific, Waltham, MA, USA) for 16 h at 37 °C was required before loading the sample in the gel electrophoresis according to the manufacturer’s protocol.

#### 2.2.4. TaqMan Analysis

The 5′ allelic discrimination TaqMan method was performed according to the manufacturer’s protocol (Thermo Fisher Scientific, Waltham, MA, USA). In short, 4.5 μL of the isolated genomic DNA (10 ng/μL) was mixed with 5 μL of 2X master mix along with 0.50 μL of a pre-designed SNP (single nucleotide polymorphism) assay in each well of a 96-well plate. Samples were analyzed through a real-time PCR system (Biorad, Hercules, CA, USA). An allelic discrimination plot was obtained for each of the measured SNPs. Heterozygous alleles containing both alleles clustered in the center of the allelic discrimination plot, whereas matched homozygous alleles clustered along the axes.

### 2.3. Statistical and Bioinformatic Analysis

#### 2.3.1. Statistical Analysis of Genetic Variability Effect

First, statistical analysis was performed by running a linear mixed model in SPSS to determine the effect of gene allele on ATNC levels, DNA adducts, and DNA strand breaks while also accounting for the study’s repeated measures and the confounding factors of sex, age, and BMI. Comparisons were made with T2 (processed red meat intervention), set as the reference, compared to T1 (baseline), T3 (white meat intervention), and T4 (PHYTOME meat). *p*-values < 0.05 were considered statistically significant and were expressed as * *p* < 0.05. Univariate analysis of variance, or more specifically, a 2-way ANOVA with Tukey post hoc testing was performed to determine if genetic variability impacted the response to the phytochemical-enriched meat if the meat was either standard or reduced nitrite. Statistical analyses were performed using IBM Statistics SPSS, version 27 (IBM, Amsterdam, The Netherlands), and Microsoft Excel 2021 (Redmond, WA, USA).

#### 2.3.2. Gene Score Computation

The gene score was calculated to evaluate the influence of each measured allele that a participant possessed on their response to each study outcome. For the gene score, machine learning was employed through R to calculate relative and scaled importance values for each gene and its alleles and for each outcome (ATNC levels, DNA adducts, and TM) for the T2 vs. T4 comparison [38]. Only gene groups of a sufficient size were included in the analysis (n > 5). Due to our smaller dataset, we were not able to split the dataset in the classical train–test strategy, as this would lead to model overfitting. Even so, the training process involved cross-validation of the entire dataset, thus reducing as much as possible the potential training bias with the data available.

The resulting model produced a list of relative importance values. These values represent the relative importance of different alleles in the model’s predictions. These values are scaled to provide a standardized measure of the impact of each allele on the model’s performance. The scaling ensures that the importance values are comparable across different alleles. Higher-scaled importance values indicate greater influence on the model’s predictions, helping to identify which alleles contribute more strongly to the model’s decision-making process. All importance values were scaled by dividing their importance value by the most important allele. This resulted in values ranging from 0 to 1 for each allele, with 1 representing the allele that was most important in predicting the outcome. The top alleles, which predicted a larger magnitude of change in an outcome variable, were then reported for each outcome variable. The resulting weighted importance values were then assigned to each participant according to their corresponding genotype, and the sum of each gene generated their gene score.

Due to the overall protective effect or non-effect observed from our linear mixed model analysis on study outcomes, a greater relative importance value would indicate a more protective allele, and a greater gene score would indicate a more protective combination of genotypes. The change in each participant’s outcome value (ATNC, adducts, and TM) from T2 to T4 was then plotted against the participants’ gene score for that outcome. The samples were split into two groups, namely, “Poor-Responders” and “Responders”, by identifying the cut-off along the gene score axis where the samples began to cluster below a null change in outcome. This cut-off was below the median for ATNC, but at the median for adducts and TM. A regression linear model was built with the genotoxic outcome variable as the dependent variable, the gene score of that genotoxic metric as the independent variable, and age, sex, and BMI as the covariates. The null hypothesis for each coefficient posited that the gene score did not affect the dependent variable. *p*-values were obtained through hypothesis testing using the t-distribution.

#### 2.3.3. Differentially Expressed Genes

Changes in gene expression were measured by Agilent 8 × 44 K whole human genome microarrays in colonic tissue in the PHYTOME study [22]. These data were deposited in NCBI’s Gene Expression Omnibus and are accessible through GEO series accession number GSE 147996. In total, 16734 genes were involved as the input file.

A list of differentially expressed genes (DEGs) for each GSTM1 allele was generated using the Linear Model for Microarray Analysis (LIMMA) analysis from Bioconductor [39] (*p* < 0.05), correcting for sex, age, BMI, and batch of microarray hybridization between the different groups. LIMMA makes use of linear models to assess differential expression in the context of multifactor-designed experiments and analyze comparisons between many RNA targets simultaneously.

#### 2.3.4. Pathway Analysis

For the identification of over-represented biological pathways, gene lists were uploaded onto the web-tool ConsensuspathDB using the gene identifier ‘gene symbol’ (HGNC symbol), (false discovery rate (FDR) corrected *p* < 0.01 and >5 genes per pathway). Next, pathways were clustered by their categorical cellular processes. A selection of processes in which most pathways occurred was explored in more detail, and their role in the development of colorectal cancer risk was investigated. Matched pathways belonging to the category ‘Disease’ were omitted due to their irrelevance in the context of a healthy study population.

## 3. Results

### 3.1. Candidate Gene Selection via Systematic Review

#### 3.1.1. Study Selection

**Figure 2:**
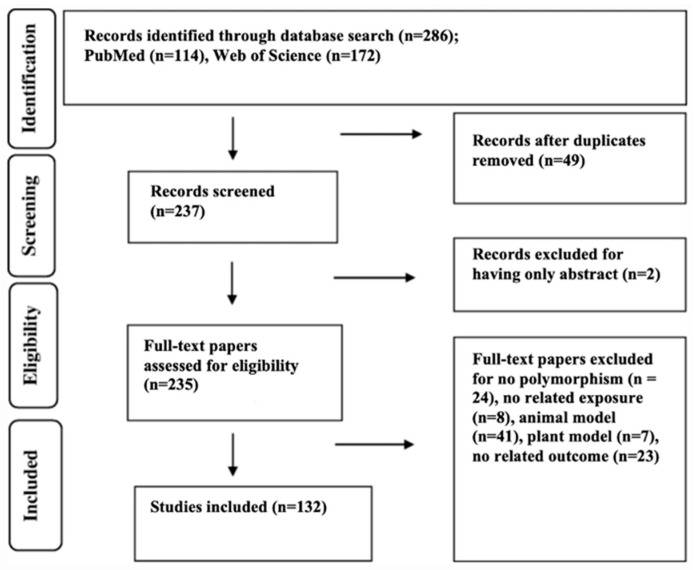
PRISMA flow diagram. Identification, screening, eligibility, and inclusion phase of the literature search.

A total of 286 studies (PubMed, n = 114; Web of Science, n = 172) were identified during the initial search process. A total of 49 records were removed due to their providing duplicated results. After the title and abstract of each study were examined, two studies were excluded for having only an abstract. This resulted in 235 studies being assessed for eligibility, where another 103 were excluded, leaving 132 included studies (Figure 2).

#### 3.1.2. Polymorphism Selection

Relevant gene polymorphisms were selected for their effect on phytochemical and meat metabolism, NOC formation, DNA damage, cell proliferation, and colorectal cancer risk. The polymorphisms were selected based on a known or expected association with ATNC, DNA adduct formation, DNA strand breaks, gene expression levels, and detoxification of xenobiotic compounds, in addition to DNA repair. The genes with variants reported most frequently in the literature were selected. The analyzed SNPs, universal ID codes, the amino acid change related to the polymorphism, enzyme function, and the expected polymorphism effect are listed (Table 3). Considering the relatively small sample size of our study, priority of selection was given to the non-synonymous SNPs of selected genes previously reported in the Dutch population. Information about the associations between the most-studied single nucleotide polymorphisms and the risk for colorectal cancer from the selected studies can be found in Table A2 in Appendix A.

### 3.2. Genetic Polymorphism Analysis

#### 3.2.1. Polymorphism Distribution in the Study Population

Using TaqMan and PCR analysis, genetic polymorphisms were identified in the PHYTOME study population. In the last three columns of this table, the frequency of wildtype, heterozygous, and variants of these polymorphisms in the current intervention population can be found (Table 3).

After the participants’ genotypes were determined, subgroup analysis of the outcome measures of the PHYTOME study (ATNC, DNA adducts, and DNA strand breaks) was performed for the different polymorphisms. For this purpose, only genetic variants with a group of at least five participants were included. Therefore, CYP1A2*1D, CYP2E1, NAT1*10, and NAT2*7, and the variants of NQO1*2 and MGMT were excluded from all statistical analyses. GSTT1*0 was also excluded from ATNC analysis due to having only four samples in the variant group.

#### 3.2.2. Effect on ATNC Levels in Fecal Water

**Figure 3:**
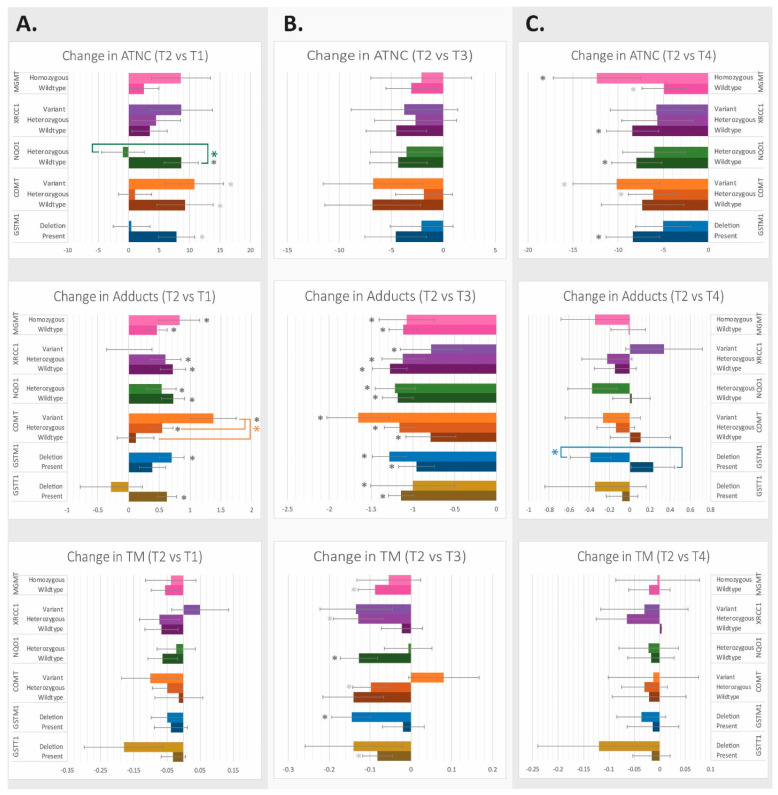
(**A**) Changes in ATNC levels, DNA adducts, and DNA strand breaks (TM) by genotype after the addition of processed red meat products to participant ad libitum daily food intake. (**B**) Changes in ATNC levels, DNA adducts, and TM by genotype after the replacement of processed red meat products with white meat. (**C**) Changes in ATNC levels, DNA adducts, and TM by genotype after the addition of phytochemical-rich natural extracts to processed red meat products. TM = tail moment. Dark gray * = the change in the outcome is significant for this allele group for the given time comparison (*p* < 0.05); if light gray *, then the change is significant only before FDR correction. Colored * = the change in the outcome is significantly different for an allele group within a genotype (*p* < 0.05). Data are expressed as means ± SEMs.

Genotype variations in NQO1 impacted changes in ATNC levels when comparing T1 vs. T2, which tested the effect of adding 300 g of processed red meat products to the participants’ baseline diet for two weeks (Figure 3). Those with the NQO1 wildtype had a significant increase in ATNC levels from their baseline levels (*p* < 0.005), which increased significantly more than in those with the NQO1 heterozygous variant (*p* < 0.05). Those with the COMT wildtype and homozygous variants and those with the GSTM1 wildtype variant also exhibited ATNC levels significantly increased from baseline (while the COMT heterozygous variant and the GSTM1 deletion variant groups did not), but these differences were not significant after FDR correction.

For the comparison of T2 vs. T3, which tests the effect of exchanging processed red meat products with white meat for two weeks, no significant differences were seen by SNP variant group, but, overall, a decrease in ATNC levels on average was observed in all samples.

Finally, for the comparison T2 vs. T4, which tests the effect of adding phytochemicals to processed red meat products vs. standard processed red meat for two weeks, four gene variant groups had a significant change in ATNC levels after FDR correction. In order of lowest *p*-values, those with the XRCC1 wildtype, the NQO1 wildtype, the GSTM1 wildtype, and the MGMT homozygote variant had the largest reduction in ATNC levels after phytochemicals were added to the meat.

#### 3.2.3. Effect on O6-methylguanine DNA Adduct Level

For T1 vs. T2, or the addition of 300 g of processed red meat products for two weeks compared to baseline, all genotype groups resulted in a significant increase in adduct levels, except for those with the GSTT1 deletion variant, the GSTM1 wildtype, the COMT wildtype, and the XRCC1 variant (Figure 3). Those with the COMT variant underwent an increase in DNA adduct levels that was significantly greater than those with the wildtype allele.

For T2 vs. T3, or the exchange of processed red meat with white meat, all genotype groups showed a decreased adduct level, with all but two gene groups significant after FDR correction. No genotype for a single gene resulted in a higher or reduced level of DNA adducts, due to the near universal response of a reduction in DNA adducts when red meat was exchanged with white. This was likely due to the removal of catalyzing heme.

For T2 vs. T4, or the addition of phytochemicals to processed red meat, a more heterogeneous response in DNA adducts was seen. Like the original results, no gene group resulted in a significant change in DNA adducts after the addition of phytochemicals. However, the responses by those with the GSTM1 deletion and present alleles were significantly different from each other. DNA adduct levels in the group with the GSTM1 deletion allele decreased by 0.38 + 0.20, whereas those with the wildtype variant increased by 0.23 + 0.21.

#### 3.2.4. Effect of Genotype on DNA Strand Breaks

After the addition of processed red meat to the participants’ baseline diet for two weeks, no significant changes were seen in ex vivo-induced DNA strand breaks (Figure 3). When the red meat was exchanged with white meat, groups with the NQO1 wildtype and GSTM1 deletion variants exhibited significantly decreased DNA strand breaks after FDR correction. After the addition of phytochemicals to red meat, there were no significant changes in tail moment (TM) by allele group.

### 3.3. Effect of Genotype on Standard- vs. Reduced-Nitrite PHYTOME Meat

**Figure 4:**
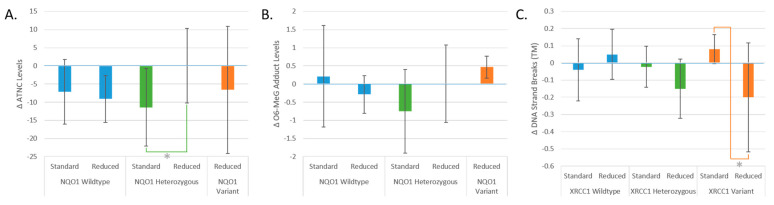
(**A**) Change in DNA adduct levels. (**B**) ATNC levels by NQO1 allele groups and whether the participants consumed standard- or reduced-nitrite phytochemical-enriched meat. (**C**) Change in DNA strand breaks (median tail moment) by XRCC1 allele groups and whether participants consumed standard- or reduced-nitrite phytochemical-enriched meat. “Standard” represents the meat that had standard-level nitrites, “Reduced” represents the meats that had reduced-nitrite levels, and “TM” is tail moment. * = *p* < 0.05. Data are expressed as means ± SEMs.

The following changes reflect the T2 vs. T4 comparison and thus the effect of ingesting processed red meat enhanced with natural extracts. DNA adduct and ATNC levels were significantly affected by the NQO1 genotype and whether the phytochemical-enriched meat consumed was standard- or reduced-nitrite (*p* = 0.04 and *p* = 0.021, respectively) (Figure 4A,B). In particular, the ATNC levels of those with the heterozygous variant were significantly different among those who had the low- versus standard-nitrite meat (*p* = 0.029). ATNC levels decreased in the NQO1 heterozygous group with the addition of phytochemicals, but no difference occurred in those who consumed the reduced-nitrite meat with this genotype. The change in DNA strand breaks in those with the XRCC1 variant was significantly different in those consuming standard- vs. reduced-nitrite meat. A reduction in DNA strand breaks occurred for those who had the XRCC1 variant allele and consumed the reduced-nitrite meat, but not in those who consumed the standard-nitrite meat (Figure 4C).

### 3.4. Protective Gene Score

**Figure 5:**
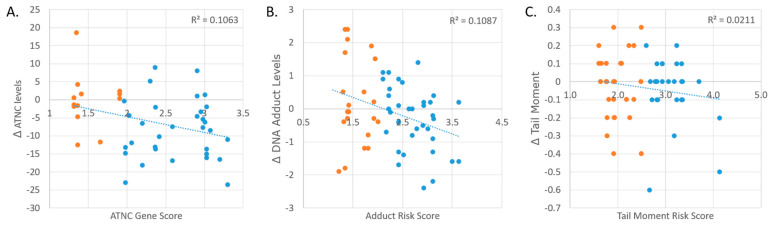
(**A**) The change in ATNC levels and the ATNC gene score of each participant sample (n = 45). (**B**) The change in DNA adduct levels and the DNA adduct gene score of each participant sample (n = 57). (**C**) The change in tail moment and the tail moment gene score of each participant sample (n = 55). These changes are from the comparison between processed red meat consumption with and without added phytochemicals (T2 vs. T4). The orange dots represent samples that were grouped by their gene score into the “Poor Responders” group, whereas the blue dots represent samples in the “Responders” group.

Five genes were used to train the machine learning model: GSTM1, NQO1, COMT, MGMT, and XRCC1. A sixth gene, GSTT1, was included for DNA adducts and tail moment. These were selected for analysis in view of their sufficient sample sizes. Of the 61 participants with full genotype data for the six genes (n = 61), there were 38 unique genotype combinations, and no genotype combination was seen more than five times in the sample population. The resulting AUC (area under the curve) values of each model were above 0.75. The relative importance variables for each allele were used to compute the gene scores for each participant (Table 4). The subsequent gene scores and the respective changes in outcomes at T2 vs. T4 accounted for 10.6%, 10.9%, and 2.1% of the variance in the participant responses in each outcome, respectively (Figure 5).

**Figure 6:**
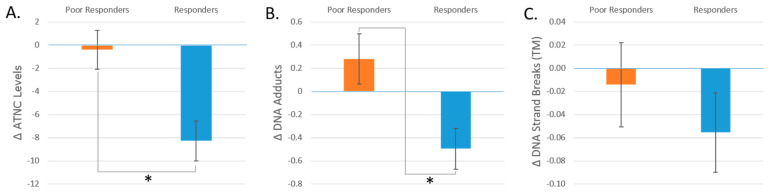
(**A**) The change in ATNC levels by gene score group. (**B**) The change in DNA adducts by gene score group. (**C**) The change in DNA strand breaks by gene score group. These changes are from the comparison between processed red meat consumption with and without added phytochemicals (T2 vs. T4). * = *p* < 0.05. Data are expressed as means ± SEMs.

The participants were split into either a “Poor Responders” or a “Responders” gene score group, with the “Responders” group containing the larger gene scores. Thus, “Poor Responders” had an ATNC gene score in the range of 1.32–1.91, whereas the “Responders” group fell in the range of 1.96–3.30. The DNA adducts “Poor Responders” group’s gene scores were between 1.09 and 2.24, and the “Responders” group’s gene scores were between 2.25 and 3.63. The TM “Poor Responders” group’s gene scores were between 1.61 and 2.60, whereas the “Responders” group’s gene scores were between 2.66 and 4.13. The Poor Responders groups showed a reduced change in the outcomes (ATNC levels, DNA adducts, and DNA strand breaks (TM)) compared to the “Responders” groups in response to the phytochemical intervention. Changes in ATNC levels and DNA adducts were significantly different for the “Responders” and “Poor Responders” gene score groups (*p* < 0.01 for both), but not for response in TM levels (*p* ≈ 0.50) (Figure 6).

The most predictive alleles for responses in the study outcomes belonged to the genes GSTM1, NQO1, and GSTT1 (Table 5). Specifically, for response in ATNC levels, the wildtype allele for GSTM1 and the wildtype allele for NQO1 indicated a stronger reduction after the addition of phytochemicals to the processed red meat. For response in DNA adducts, a stronger reduction after the intervention was found in those with the GSTM1 variant/deletion allele and the heterozygous allele for NQO1 (the variant was not compared due to insufficient sample size). And, finally, a stronger response in DNA strand breaks was more expected in those with the GSTM1 variant/deletion allele and the GSTT1 variant/deletion allele.

### 3.5. Gene Expression and Pathway Analysis

#### 3.5.1. DEGs for GSTM1 Genotype Groups at T2 vs. T4

Differentially expressed genes (DEGs) after consumption of different meat interventions were identified by LIMMA analysis. Given the influence of GSTM1 on all of the study outcomes and the even population distribution among the two alleles, this gene was selected for further analysis. For this purpose, individuals with the GSTM1 wildtype allele were compared to individuals with the deletion variant in this gene. Because of the interest in the protective effects of adding phytochemicals to processed red meat, the comparison between T4 and T2 was evaluated.

DEGs were identified for the GSTM1 wildtype and the GSTM1 deletion variant for the comparison T2 vs. T4 on DNA adduct levels. DEGs were selected based on several criteria, including fold change (FC), *p*-value, and adjusted *p*-value (Table 6).

A higher number of DEGs was identified based on *p*-value when compared to the criterion of fold change. However, no DEGs were below the adjusted *p*-value. DEGs based on *p*-value were selected to continue in the pathway analysis for an exploratory analysis.

The expression levels of several genes were altered and specific to either the GSTM1 wildtype group or the GSTM1 variant (deletion) group. Unique to the GSTM1 wildtype group, 326 genes were upregulated and 972 genes were downregulated, whereas 220 genes were uniquely upregulated in the GSTM1 variant group and 65 were downregulated. Few overlapping genes exist. Interestingly, a few genes were up- or downregulated in both allele groups but had opposite directionality. Of these, 14 genes were found to be upregulated in the variant group but downregulated in the wildtype group. Also, three genes were found to be downregulated in the wildtype group and upregulated in the variant group (Table 7). A list of all DEGs can be found in a GitHub (San Francisco, CA, USA) repository [40].

#### 3.5.2. Pathway Analysis of DEGs

DEGs with a *p* < 0.05 were uploaded onto ConsensuspathDB and over-represented biological pathways were identified. The minimum overlap with the input list was cut off at a minimum of five genes. Over-represented biological pathways were clustered on the given cellular processes for the GSTM1 deletion and variant genotype. A list of all over-represented pathways, per genotype, can be found in a GitHub repository [40].

For the deletion variant of GSTM1, 188 genes (70.7%) from the differentially expressed genes are present in at least one pathway. In total, 26 enriched pathways were found. Involved biological processes with the most-enriched pathways were found in ‘mitotic’ and ‘signaling’ enriched pathway-based sets. For ‘mitotic,’ five enriched pathways were found, whereas seven pathways were enriched for ‘signaling,’ as can be found in Table 8. A larger number of 899 genes (73.0%) were found in at least one pathway for the GSTM1 wildtype allele. Multiple biological processes were involved in the over-represented pathways, including ‘disease,’ ‘infection,’ ‘transcription,’ ‘translation’, and ‘signaling’ (Table 9). Interestingly, ‘cell cycle’ and ‘metabolism’ pathways were also found to be over-represented in these individuals. An overview of all pathways along with the gene members present in each pathway can be found in a GitHub repository [40].

## 4. Discussion

In this study, we evaluated the effect of genetic variability on the health impact of different meat interventions aimed at reducing the risk of colorectal cancer in the PHYTOME study. The main finding in this follow-up study was that the responses in genotoxic biomarkers after the consumption of different meat interventions were partially attributable to genetic variability among the participants. This discussion mostly focuses on two comparisons. The first comparison aims to establish the effect of adding phytochemicals to processed red meat products by comparing the test day after processed red meat was consumed (T2) and the test day after processed red meat with added phytochemical extracts was consumed (T4). The second comparison aims to establish the effect of increased meat consumption by comparing the outcomes of the test day after processed red meat was consumed for two weeks (T2) and the participants’ baseline test day.

A systematic literature review was conducted to identify relevant polymorphisms that potentially influence the metabolism of phytochemicals and responses to oxidative stress and DNA damage. The PICOS and PRISMA guidelines were utilized to generate a quality list of relevant genes, some of which were indeed found to impact our study outcomes [41]. This list can be utilized in future nutrigenomic intervention studies aimed at reducing colorectal cancer risk. Despite the selection criterion for polymorphisms with a prevalence of at least 20% in the population, some polymorphisms were less prevalent in our study population. This is most likely explained by the fact that our study population was too small to detect several variants of a polymorphism. There is also the case that some polymorphisms are more prevalent in certain subgroups [42] and that results in the literature vary [43]. A larger study population could have prevented this problem, but the complex design of the study did not allow for that.

Individuals with the NQO1 wildtype allele had a significantly larger increase in fecal NOCs after consuming 300 g per day of processed red meat products for two weeks. Those with the heterozygous allele, on the contrary, did not experience an increase in these potential carcinogenic compounds. NQO1 (NAD(P)H quinone dehydrogenase (1) is an enzyme that plays a critical role in cellular protection, detoxification, and metabolism of a variety of compounds [44]. While it would be expected that those with the wildtype version of the NQO1 gene would have additional protection from endogenous formation and therefore excretion of NOCs from processed red meat, this was not seen in our study. NQO1 has recently been shown to have a binding site that can bind substrates that have two redox centers, like the two nitrogen atoms in some NOCs, leading to futile redox cycling [45]. This unproductive redox cycling leads to adverse metabolic conditions leading to negative outcomes like the generation of reactive oxygen species, but this has not been directly studied with NOCs. More research into this potential mechanism is warranted. Aligned with this, when phytochemicals were added, it was the NQO1 wildtype group that showed a more protective effect and a significant reduction in ATNC levels, whereas the heterozygous allele did not significantly change. Overall, the NOC levels in those with the NQO1 wildtype allele were affected more by the dietary interventions, while those with the heterozygous allele did not experience a notable change in NOCs after either intervention. This may be due to the unproductive redox cycling characteristic of the NQO1 enzyme formed by the NQO1 wildtype gene, leading to more harmful effects in the presence of NOCs, which is then more benefited by the phytochemical intervention, but this is still not well understood within this context.

Those with the COMT homozygous variant had the largest increase in DNA adducts after the consumption of processed red meat compared to baseline. COMT (catechol-O-methyltransferase) is an enzyme responsible for the methylation of catechol compounds, making these endogenous or exogenous compounds water-soluble and aiding in their excretion from the body. This detoxification of potentially harmful compounds prevents their accumulation in the body, which could otherwise lead to DNA damage and the formation of adducts [46]. This variant, leading to a low activity form of COMT, has been widely studied regarding its association with cancer [47]. In contrast to the heterozygous group, the COMT wildtype group did not experience an increase in DNA adducts following this intervention, suggesting a potential role of sufficient COMT enzyme activity in protecting colonic epithelial cells from damage induced by increased and prolonged processed red meat consumption.

A differing response for the COMT genotype was seen after the consumption of PHYTOME meat compared to the standard red meat intervention. For both changes in ATNC levels and DNA adducts, the wildtype group had the smallest response to the added phytochemicals, whereas those with the homozygous variant experienced the largest reduction in ATNC levels and DNA adducts. This is likely due to the known ability of COMT to metabolize polyphenols. A study of the COMT genotype and green-tea drinkers found that men with the low-activity homozygous variant retained more tea polyphenols than those with the heterozygous variant or wildtype allele, likely deriving a greater benefit from them [48]. Overall, those with the COMT homozygous variant were more responsive than the wildtype group to both interventions and especially more prone to DNA adduct formation after consuming processed red meat. Based on this evidence, it could be argued that those with the COMT wildtype allele are less at risk of the genotoxic effects of consuming processed red meats and that the added phytochemical-rich extracts do not benefit them as much because their COMT enzyme contributes to their fast breakdown, whereas those with the COMT variant allele are more at risk when consuming processed red meat and also benefit more when phytochemical extracts are added to that meat.

The GSTM1 genotype was the most pronounced in impacting the response in DNA adduct levels after the addition of the natural extracts. GSTM1 (glutathione s-transferase m1) is an enzyme that plays a role in the detoxification of chemicals, primarily by conjugating electrophilic compounds with the endogenous antioxidant glutathione, making them water-soluble and easier to eliminate from the body. This enzyme’s functional role in neutralizing potentially harmful compounds can be seen when looking at the T1 vs. T2 comparison, with the addition of processed red meat compared to baseline. Those with the GSTM1 wildtype allele did not show a significant increase in DNA adducts after this intervention, whereas those with the deletion did show a significant increase compared to baseline. In line with our results, the deficiency in GSTM1 caused by the null genotype has previously been associated with increased DNA adducts [43,49]. However, the effect of the GSTM1 genotype on DNA adducts, as stated previously, was most pronounced when phytochemicals were added to the intervention. Those with the deletion had a significantly more protective response to the dietary intervention than those with the wildtype allele. Like COMT, the GSTM1 enzyme also metabolizes phytochemicals such as isothiocyanates, and GSTM1 polymorphisms may impact their clearance and duration [34,50]. However, some studies show that phytochemical excretion is higher in those with the deletion polymorphism, suggesting a reduced effect [34]. Another explanation for the lower levels of DNA adducts after consuming PHYTOME meat in the GTM1 deletion group is that the added phytochemicals in the intervention exerted antioxidant and radical neutralizing effects which were more beneficial to those who lack this detoxifying enzyme.

From what is known in the literature, higher DNA strand break levels would be expected from increased processed red meat intake [13,51]. However, for each intervention comparison, the overall change in strand breaks measured by tail moment was too small in this study to observe sub-group effects. Therefore, no conclusions can be drawn about the effect of genotype on ex vivo-induced strand breaks after ingestion of processed meat or the addition of phytochemicals to that meat. There was, however, a significant reduction in strand breaks for those with the XRCC1 variant who consumed the PHYTOME meat with a reduced nitrite level compared to those with the variant consuming the standard meat. XRCC1 (X-ray repair cross-complementing group (1) acts as a scaffolding protein that interacts with multiple repair enzymes that allow for the repair of oxidative DNA damage and single-strand breaks [51]. Polymorphisms in the XRCC1 gene have been linked to various cancers due to this reduced DNA repair ability [52]. From our study, those with the XRCC1 variant benefit more from consuming meats that are also reduced in nitrite, beyond just compensating for the potentially deleterious compounds with added protective compounds.

To consider that multiple genes and enzymes shape biological outcomes, a protective gene score was computed for each participant in the context of the T2 vs. T4 (the addition of phytochemical extracts to processed red meat) comparison. The gene score correlated well with all outcomes when the study population was split into high and low scorers (into “Responders” and “Poor Responders” groups, respectively). The gene scores for ATNC levels and DNA adducts contributed significantly to differentiate between Responders and Poor Responders to the PHYTOME meat. The DNA adducts group had a slightly larger sample size than the others but also had the most heterogeneous response in the T2 vs. T4 comparison. For the latter reason, we believe that our model was most able to detect the different responders.

The participants with the most favorable response in levels of NOCs were those with the GSTM1 and NQO1 wildtype alleles. This contrasts with those with the largest reduction in DNA adduct levels after consuming the PHYTOME meat, the GSTM1 variant and NQO1 heterozygous groups. The formation of harmful compounds like NOCs and whether these compounds result in DNA damage or adducts seem to be differently modulated. As far as the intervention’s ability to reduce colorectal cancer risk by reducing damage to cellular DNA is concerned, those with the GSTM1, NQO1, and GSTT1 variants are more likely to benefit from consuming PHYTOME meat over standard processed red meat products.

Due to the clear influence of GSTM1 on the study outcomes for the T2 vs. T4 comparison, these allele groups were selected for further gene expression analysis to help explain the mechanisms behind these differing responses. No genes appeared as significantly differentially expressed after applying the fold-change and adjusted *p*-value threshold, so a *p*-value threshold of 0.05 was applied for exploratory analysis of potential mechanisms and generating hypotheses [53,54].

After the LIMMA analysis, 349 differentially expressed genes (DEGs) were identified for the GSTM1 variant, while the GSTM1 wildtype resulted in 1420 DEGs. Pathway analysis of the DEGs was carried out separately for the GSTM1 wildtype and the GSTM1 variant genotype groups. For the GSTM1 variant, the enriched pathways were involved in ‘mitotic processes’ and in ‘signaling’ (Table 5). The overlapping genes in the ‘mitotic processes’ involve the following genes: CEP164, TUBB4B, HAUS2, TUBG1, NEDD1, TUBA4A, and YWHAG. Besides NEDD1, all the other genes were found to be upregulated for the GSTM1 variant. In the research of Tillemant et al. (2009), NEDD1 gene was proposed as an important target for inducing cell cycle arrest [55]. The inhibition of mitosis has been identified as one of the biological activities at the molecular level for the anti-carcinogenic effect of the phytochemicals [56]. In this research, lower DNA adduct levels were found for the GSTM1 variant for the comparison of T4 and T2. Decreased mitotic pathways or the induction of cell cycle arrest to potentially allow for sufficient DNA damage repair in these participants are now suggested to contribute to this effect. This is in accordance with the research of Uusküla et al. 1995, where the GSTM1 null genotype was also associated with decreased mitotic processes [57].

For the GSTM1 wildtype, more enriched pathways were found which were also affecting a broader spectrum of biological functions, such as pathways involved in ‘disease,’ ‘infection,’ ‘transcription,’ ‘translation’, and ‘signaling’ (Table 6). Interestingly, there were also altered pathways involved in ‘cell cycle’ and in ‘metabolism.’ Regarding phytochemicals, the pathway ‘metabolism of water-soluble vitamins and cofactors’ was found to be enriched in the GSTM1 wildtype individuals. It could thus be hypothesized that the added natural extracts in the PHYTOME meat were metabolized faster and could only exert their beneficial action to a lesser extent, resulting in about the same DNA adduct levels as compared to the processed red meat intervention.

The vitamin D receptor pathway was also enriched in the GSTM1 wildtype group (Table 6). The genes involved in this pathway include TGFB1 and ABCB1. Due to the activation of the vitamin D receptor in this pathway, transcription factors for various biological processes, including cellular differentiation and immune response, are activated. These physiological alterations may explain the relationship between sufficient vitamin D status and reduced risk of colorectal cancer mortality [58]. In our research, TGFB1 was downregulated in this pathway for the GSTM1 wildtype. This gene has a growth inhibitory effect and therefore exerts a reduction in cancer risk [59]. The lower DNA adduct levels for the GSTM1 wildtype group compared to the variant after the consumption of processed red meat compared to baseline could be explained through this mechanism.

Another essential component of this pathway is the ABCB1 gene, an ATP-binding cassette transport protein crucial for the transcellular movement of phytochemicals and xenobiotic compounds across the intestinal epithelium. This transporter plays a pivotal role in determining bioavailability following oral intake. It can efflux various conjugated and unconjugated substances from intestinal cells, shuttling them either towards the basolateral blood side, thereby facilitating absorption, or back into the intestinal lumen, consequently reducing bioavailability [60,61]. We postulate that the upregulated activity of the ABCB1 gene, responsible for transporting phytochemicals back into the intestinal lumen, may contribute to reduced phytochemical absorption in those with the GSTM1 wildtype allele, thus contributing to the lack of change in DNA adduct levels.

Also of interest, the “Metabolism of water-soluble vitamins and cofactors” pathway exhibited over-representation in the GSTM1 wildtype group. A gene associated with this pathway is GSTO1, which represents another isoform of the glutathione-S-transferase genes (GST). Like GSTM1 and GSTT1, GSTO1 functions as a phase II metabolizing enzyme. These enzymes play a pivotal role in metabolizing a broad spectrum of environmental chemicals, including carcinogens, drugs, and phytochemicals [56,62]. Given the upregulation of the GSTO1 gene in our differential gene expression analysis, it can be inferred that phytochemicals are more efficiently metabolized within this pathway. The augmented activity of phase II metabolizing enzyme pathways in individuals with the GSTM1 wildtype allele likely contributes to the lack of impact that the added phytochemicals had on reducing DNA adduct levels in this group.

Ultimately, several genetic variants were found to influence the responses to different meat interventions. To the best of our knowledge, no other studies have been performed indicating this effect or predicting inter-individual responses to these specific dietary intervention products. Hence, more research is needed in this context. Expanding DEG and pathway analysis beyond the GSTM1 gene for a single time comparison and exploring other genes, outcome measures, and comparisons is warranted. Furthermore, assessing protein levels associated with the genes within a relevant pathway is valuable given that DNA gene expression does not perfectly predict protein levels, enhancing precision in our understanding of the mechanisms at play [63].

In summary, this study identified a list of potentially relevant gene polymorphisms associated with the inter-individual risk of colorectal cancer by means of a systematic literature search. These SNPs included COMT, CYP1A2, CYP2E1, GSTM1, GSTT1, MGMT, NAT1, NAT2, NQO1, and XRCC1. In this follow-up research, we observed the interaction of these gene polymorphisms (COMT, GSTM1, GSTT1, MGMT, NQO1, and XRCC1) with different meat diets and on outcomes linked to colorectal cancer risk: ATNC levels, DNA adduct levels, and DNA strand breaks. We also predicted the most impactful gene alleles affecting these responses, identifying individuals who might be the most protected against colorectal cancer risk by the addition of phytochemical-rich natural extracts to their processed red meat products. In particular, the GSTM1, GSTT1, and NQO1 variants most impacted the response in these outcomes. Furthermore, we discovered DEGs from colon tissue of the GSTM1 wildtype and variant individuals following the consumption of PHYTOME meat versus standard processed red meat products. These genes revealed over-expressed pathways, shedding light on potential mechanistic variations in the colons of individuals with a specific genotype after consuming a diet aimed at reducing colorectal cancer risk. In particular, pathways relating to cell cycle arrest and phytochemical and vitamin D metabolism may play a role in GSTM1 wildtype resilience against the potentially deleterious effects of processed red meat consumption.

Our findings hold valuable implications for dietitians in formulating personalized dietary recommendations to reduce colorectal cancer risk, taking an individual’s genetic makeup into consideration. For instance, individuals with the COMT homozygous variant may be at a higher risk of DNA adduct formation after consuming processed red meat. Therefore, dietitians could recommend that these individuals remove or minimize processed red meat consumption. On the other hand, individuals with the GSTM1 variant (deletion) allele may derive more substantial benefits from incorporating polyphenol-rich foods like green tea, white grape, and rosemary into their diets when consuming processed red meat. Dietitians can play a crucial role in encouraging the inclusion of these foods to help mitigate the associated risks. Furthermore, our study suggests that producers of processed meats could explore alternatives to nitrite, such as the plant extracts used in our intervention. Previous research has indicated that these extracts can reduce excreted ATNC levels in comparison to traditionally processed red meats [22]. Therefore, this substitution may offer a healthier option for consumers concerned about colorectal cancer risk. This research represents a significant stride in the realms of nutrigenomics and personalized nutrition, as it identifies genes that modulate the levels of biomarkers and phenotypic markers in response to the consumption of different meat products, thereby advancing the mechanistic understanding needed to formulate individualized dietary recommendations.

## 5. Conclusions

In conclusion, our study examines the relationship between relevant genetic polymorphisms and meat interventions in modulating genotoxic biomarkers associated with colorectal cancer risk. Genetic polymorphisms, such as those in COMT, GSTM1, GSTT1, NQO1, and XRCC1, were identified as significant factors influencing individual responses to different meat interventions. Notably, GSTM1, NQO1, and GSTT1 genotype emerged as the most relevant factors affecting the change in participant genotoxic biomarkers in response to the addition of phytochemical-rich plant extracts to processed red meat (PHYTOME meat). These findings underscore the importance of considering genetic makeup when formulating dietary recommendations to mitigate colorectal cancer risk. Furthermore, our study proposes hypotheses for the mechanistic variations associated with GSTM1 genotype in response to the PHYTOME meat versus standard processed red meat. Gene expression analysis revealed potential pathways, including cell cycle regulation, phytochemical metabolism, and vitamin D receptor signaling, that may contribute to the observed differences in genotoxic biomarker responses. These insights open avenues for future research in nutrigenomics and personalized nutrition, ultimately aiding in the development of more effective strategies for reducing colorectal cancer risk and promoting overall health.

## Figures and Tables

**Table 1:** PHYTOME meat product formulations: levels of nitrite and nitrate (mg/kg) and natural extracts (g/kg) added during meat manufacturing.

Meat	Added Nitrite/Nitrate (mg/kg)		Natural Extracts (g/kg) ^(a)^						
	Standard-Nitrite PHYTOME Meat	Reduced-Nitrite PHYTOME Meat	Standard-Nitrite and Reduced-Nitrite PHYTOME Meat						
			Polygonum	Rutin/Sophora	Green Tea	Origanox	White Grape	Rosemary	Acerola
Cooked ham	100/0	25/0	0.1	0	0	0	0	0	2.5
Raw ham	150/150	75/0	0.3	0	1.5	0.75	0.75	0.3	2.2
Cooked sausage	150/0	25/0	0.1	0.5	1.2	0.65	0.65	0.65	2.5
Dry sausage	150/150	25/0	0.05	0.25	0.65	0.65	0.65	0.65	2.5
Dry cured ham	150/150	0/0	0.08	0.4	1.25	1.25	1.25	1.25	1.25
Dry sausage Southern style	80/150	0/0	0.05	0.25	0.65	0.65	0.65	0.65	2.5

^(a)^ Botanic source or trade name, main bioactive molecule composition %, supplier: Polygonum Cuspidatum root, Resveratrol 98%, Nutraceutica, Italy; Sophora Japonica, Rutin 98%, Indena, Italy; Green tea, Epigallocatechin gallate (EGCG) 40%, Indena, Italy; Origanox WS-T, Polyphenols 30% as gallic acid from oregano, sage, Melissa, Frutarom, Italy; White grape NutriPhy, Polyphenols 95% as gallic acid, Chr Hansen, Italy; Rosemary—Aquarox Polyphenols 15% as gallic acid, Vitiva, Slovenia; Acerola, ascorbic acid 17%, Raps, Germany.

**Table 2:** List of genes for PCR analysis, their forward and reverse primers, and product size.

Gene	Primer Forward	Primer Reverse	Product Size (bp)
β-globin	5′-CAACTTCATCCACGTTCACC-3′	5′-GAAGAG CCAAGGACAGGTAC-3′	268
CYP2E1	5′-GTGATGGAAGCCTGAAGAACA-3′	5′-CTTTGGTGGGGTGAGAACAG-3′	729 (with insertion)
			633 (without insertion)
GSTM1*0	5′-GAACTCCCTGAAAAGCTAA AGC-3′	5′-GTTGGGCTCAAATATACGGTGG-3′	215
GSTT1*0	5′-TTCCTT ACTGGTCCTCACATCTC-3′	5′-TCACCGGATCATGGCCAGCA-3′	480
MGMT	5′-TGCAGGACCACTCGAGGCTGCCA-3′	5′-CCCGGATATGCTGGGACAGCCC-3′	167 (A allele)
			97 and 70 (G allele)

**Table 3:** List of SNPs, their position, and the amino acid change that is related to the polymorphism.

SNP	Amino Acid Change	dbSNP ID	Function	Effect on Enzymatic Function	Frequencies ^a^			
					wt	hz	v	nr
COMT*1	V158M	Rs4680	Phase II detoxification	Decreased enzyme activity	14	39	10	-
CYP1A2*1D	−2467T/delT	Rs35694136	Phase I bioactivation	Increased enzyme activity	58	0	2	2
CYP2E1	96-bp insertion	Rs28371744	Phase I bioactivation	Increased enzyme activity	59	2	0	2
GSTM1*0	Deletion		Phase II detoxification	No enzyme activity	30	-	32	2
GSTT1*0	Deletion		Phase II detoxification	No enzyme activity	57	-	5	1
MGMT	c.-56C>T	Rs16906252	DNA repair	Decreased enzyme activity	44	13	1	5
NAT1*10	S214A	Rs4986783	Phase II detoxification	Decreased enzyme activity	60	3	0	-
NAT2*7	G286E	Rs1799931	Phase II detoxification	Decreased enzyme activity	59	4	0	-
NQO1*2	P187S	Rs1800566	Phase II detoxification	Decreased enzyme activity	37	23	3	-
XRCC1*4	Q399R	Rs25487	DNA repair	Decreased enzyme activity	30	23	10	-

^a^ wt = homozygous wildtype, hz = heterozygous, v = homozygous variant, nr = no result. The numbers reflect the number of subjects carrying that genotype. A hyphen indicates that the method was not able to distinguish between heterozygous or homozygous wildtype. Deletion variants were also listed under “v”.

**Table 4:** Relative importance variables for each allele and each outcome variable. wt = homozygous wildtype, hz = heterozygous, v = homozygous variant or deletion.

ATNC			Adducts			Tail Moment		
Gene	Genotype	Importance Variable	Gene	Genotype	Importance Variable	Gene	Genotype	Importance Variable
GSTM1	wt	1	GSTM1	v	1	GSTM1	v	1
NQO1	wt	0.831808925	NQO1	hz	0.854708135	GSTT1	v	0.89053607
MGMT	v	0.646299422	GSTT1	v	0.753620148	MGMT	wt	0.725150943
COMT	v	0.489445686	MGMT	v	0.694708467	NQO1	hz	0.709075689
COMT	hz	0.439706266	XRCC1	hz	0.383528173	MGMT	hz	0.572865427
XRCC1	wt	0.402637959	XRCC1	wt	0.364398777	COMT	hz	0.437153459
COMT	wt	0.393060923	COMT	hz	0.33453232	XRCC1	hz	0.371162295
XRCC1	v	0.384819269	XRCC1	v	0.314071953	XRCC1	wt	0.354943871
MGMT	wt	0.353700578	MGMT	wt	0.305291533	COMT	wt	0.335979998
XRCC1	hz	0.281867206	GSTT1	wt	0.246379852	NQO1	wt	0.290924311
NQO1	hz	0.168191075	COMT	v	0.238555729	XRCC1	v	0.198945999
GSTM1	v	0	COMT	wt	0.154783726	COMT	v	0.162921727
			NQO1	wt	0.145291865	GSTT1	wt	0.10946393
			GSTM1	wt	0	GSTM1	wt	0

**Table 5:** The most predictive alleles for study outcome responses for T2 vs. T4.

	Gene	Genotype
Largest Reductions in ATNC levels	GSTM1	Wildtype/Present
	NQO1	Wildtype (G/G)
Largest Reductions in DNA adducts	GSTM1	Variant/Deletion
	NQO1	Heterozygous (G/A)
Largest Reductions in DNA strand breaks	GSTM1	Variant/Deletion
	GSTT1	Variant/Deletion

**Table 6:** DEG analysis results of microarray expression data.

Comparison T2 vs. T4	Variant GSTM1	Wildtype GSTM1
|FC| ≥ 1.2	302	424
Upregulated	54	73
Downregulated	248	351
*p* value < 0.05	349	1420
Adj. *p* value < 0.2	0	0
|FC| and *p* value	43	255
|FC| and adj. *p* value	0	0

**Table 7:** List of differentially expressed genes (*p* < 0.05) for each allele group of GSTM1 for the comparison of T2 vs. T4, where the direction of expression is the opposite of the other allele group. FC = fold change.

		GSTM1 Wildtype (Present)			GSTM1 Variant (Deletion)		
Gene Symbol	Gene Name	Direction of Expression	log FC	*p*-Value	Direction of Expression	log FC	*p*-Value
FUBP1	Far upstream element (FUSE) binding protein 1	**↓**	−0.103	4.55 × 10^−2^	**↑**	0.091	4.87 × 10^−2^
PREB	Prolactin regulatory element binding	**↓**	−0.103	2.82 × 10^−2^	**↑**	0.098	3.38 × 10^−2^
COG8	Component of oligomeric golgi complex 8	**↓**	−0.123	4.32 × 10^−2^	**↑**	0.120	3.22 × 10^−2^
AMER1	APC membrane recruitment protein 1	**↓**	−0.188	4.80 × 10^−3^	**↑**	0.129	4.86 × 10^−2^
TANGO6	Transport and golgi organization 6 homolog	**↓**	−0.264	3.51 × 10^−2^	**↑**	0.134	2.53 × 10^−2^
IL1RAP	Interleukin 1 receptor accessory protein	**↓**	−0.206	6.14 × 10^−3^	**↑**	0.137	4.70 × 10^−2^
FAM203A	Family with sequence similarity 203, member A	**↓**	−0.134	1.40 × 10^−2^	**↑**	0.150	1.30 × 10^−2^
TOMM40	Translocase of outer mitochondrial membrane 40 homolog	**↓**	−0.207	2.66 × 10^−2^	**↑**	0.160	3.69 × 10^−2^
SNRPE	Small nuclear ribonucleoprotein polypeptide E	**↓**	−0.136	1.77 × 10^−2^	**↑**	0.163	4.92 × 10^−2^
COQ3	Coenzyme Q3 methyltransferase	**↓**	−0.216	1.11 × 10^−3^	**↑**	0.163	3.65 × 10^−2^
HEATR3	HEAT repeat containing 3	**↓**	−0.151	3.06 × 10^−2^	**↑**	0.168	3.28 × 10^−2^
NOP2	NOP2 nucleolar protein	**↓**	−0.204	4.13 × 10^−3^	**↑**	0.172	3.91 × 10^−2^
GZF1	GDNF-inducible zinc finger protein 1	**↓**	−0.157	2.95 × 10^−2^	**↑**	0.223	3.04 × 10^−3^
PPFIA3	Protein tyrosine phosphatase, receptor type, f polypeptide (PTPRF), interacting protein (liprin), alpha 3	**↓**	−0.153	2.20 × 10^−2^	**↑**	0.246	8.77 × 10^−3^
FBXO32	F-box protein 32	**↓**	0.238	3.44 × 10^−2^	**↑**	−0.248	3.07 × 10^−2^
CBFA2T2	Core-binding factor, runt domain, alpha subunit 2; translocated to, 2	**↓**	0.123	3.60 × 10^−2^	**↑**	−0.154	3.08 × 10^−2^
SLMAP	Sarcolemma associated protein	**↓**	0.089	4.44 × 10^−2^	**↑**	−0.117	4.61 × 10^−2^

**Table 8:** Over-represented pathway-based sets for GSTM1 deletion variant.

Biological Process	Pathway
Mitotic	Loss of Nlp from mitotic centrosomes
	Recruitment of NuMA to mitotic centrosomes
	Recruitment of mitotic centrosome proteins and complexes
	Mitotic G2-G2/M phases
	Mitotic prometaphase
Signaling	FoxO family signaling
	FoxO signaling pathway—Homo sapiens (human)
	MAPK signaling pathway—Homo sapiens (human)
	Prolactin signaling pathway
	mTOR signaling pathway—Homo sapiens (human)
	Cytokine signaling in immune system
	MAPK signaling pathway

**Table 9:** Enriched pathway-based sets for GSTM1 wildtype variant.

Biological Process	Pathway
Activation	Validated targets of C-MYC transcriptional activation
	Activation of csk by camp-dependent protein kinase inhibits signaling through the t cell receptor
	GPVI-mediated activation cascade
	Activation of the mRNA upon binding of the cap-binding complex and eIFs and subsequent binding to 43S
	Activation of camp-dependent protein kinase pka
Cell Cycle	Cell cycle—Homo sapiens (human)
	Cell cycle
	G1 to S cell cycle control
	TP53 regulates transcription of cell cycle genes
	Cyclins and cell cycle regulation
	TP53 regulates transcription of genes involved in G2 cell cycle arrest
	RHO GTPase cycle
	RHOBTB GTPase cycle
	RHOBTB1 GTPase cycle
	RHOBTB2 GTPase cycle
	HIV life cycle
Deficiency	Response of EIF2AK4 (GCN2) to amino acid deficiency
	S-Adenosylhomocysteine (SAH) Hydrolase deficiency
	Methionine Adenosyltransferase deficiency
	Glycine N-methyltransferase deficiency
	Methylenetetrahydrofolate Reductase deficiency (MTHFRD)
	Cystathionine Beta-Synthase deficiency
	Purine Nucleoside Phosphorylase deficiency
	Xanthine Dehydrogenase deficiency (Xanthinuria)
	Adenylosuccinate Lyase deficiency
	Adenine phosphoribosyltransferase deficiency (APRT)
	Myoadenylate deaminase deficiency
	Molybdenum Cofactor deficiency
	Adenosine Deaminase deficiency
Immune response	CD4 T cell receptor signaling-JNK cascade
	CD4 T cell receptor signaling-NFkB cascade
	CD4 T cell receptor signaling-ERK cascade
	GPVI-mediated activation cascade
	Caspase cascade in apoptosis
	Caspase cascade in apoptosis
Infection	Epstein-Barr virus infection—Homo sapiens (human)
	Human T-cell leukemia virus 1 infection—Homo sapiens (human)
	Staphylococcus aureus infection—Homo sapiens (human)
	Infection with Mycobacterium tuberculosis
	Influenza infection
	Human cytomegalovirus infection—Homo sapiens (human)
	HIV infection
	Kaposi sarcoma-associated herpesvirus infection—Homo sapiens (human)
Metabolism	Metabolism of RNA
	Selenoamino acid metabolism
	Metabolism of non-coding RNA
	Metabolism of proteins
	Folate metabolism
	NO metabolism in cystic fibrosis
	Methionine metabolism
	Lysine metabolism
	One-carbon metabolism
	NAD metabolism in oncogene-induced senescence and mitochondrial dysfunction-associated senescence
	Purine metabolism
	Metabolism of water-soluble vitamins and cofactors
	Metabolism of amino acids and derivatives
	Glutathione metabolism—Homo sapiens (human)
	Metabolism of folate and pterines
	Etoposide metabolism pathways
	Pyrimidine metabolism
Processing	Antigen processing and presentation—Homo sapiens (human)
	Processing of Capped Intron-Containing Pre-mRNA
	tRNA processing in the nucleus and cytosol
	rRNA processing
	mRNA processing
	Processing of Capped Intronless Pre-mRNA
Receptor	CD4 T cell receptor signaling-JNK cascade
	B Cell Receptor signaling pathway
	CD4 T cell receptor signaling-NFkB cascade
	CD4 T cell receptor signaling-ERK cascade
	Activation of csk by camp-dependent protein kinase inhibits signaling through the t cell receptor
	CD4 T cell receptor signaling
	Fc-epsilon receptor I signaling in mast cells
	B cell receptor signaling pathway—Homo sapiens (human)
	Viral protein interaction with cytokine and cytokine receptor—Homo sapiens
	Kit receptor signaling pathway
	Cystic fibrosis transmembrane conductance regulator (cftr) and beta 2 adrenergic receptor (b2ar) pathway
	Vitamin D Receptor pathway
Regulation	Transcriptional regulation of granulopoiesis
	Cyclins and cell cycle regulation
	chrebp regulation by carbohydrates and camp
	Regulation of RhoA activity
	Regulation of KIT signaling
	Transcriptional regulation by E2F6
	Regulation of TP53 activity through methylation
Signaling	PD-1 signaling
	B Cell Receptor signaling pathway
	TCR signaling
	P53 signaling pathway
	Fc epsilon RI signaling pathway—Homo sapiens (human)
	Downstream TCR signaling
	Activation of csk by camp-dependent protein kinase inhibits signaling through the t cell receptor
	CD4 T cell receptor signaling
	IL12-mediated signaling events
	Signaling by SCF-KIT
	Interferon signaling
	Fc-epsilon receptor I signaling in mast cells
	Cytokine signaling in immune system
	B cell receptor signaling pathway—Homo sapiens (human)
	Chemokine signaling pathway—Homo sapiens (human)
	NF-kappa B signaling pathway—Homo sapiens (human)
	Chemokine signaling pathway
	Kit receptor signaling pathway
	Notch signaling pathway Netpath
	Photodynamic therapy-induced HIF-1 survival signaling
	Interferon type I signaling pathways
	FoxO signaling pathway—Homo sapiens (human)
	IL-18 signaling pathway
	Regulation of KIT signaling
	Signaling by Rho GTPases, Miro GTPases, and RHOBTB3
	Glioblastoma signaling pathways
	Signaling by Rho GTPases
	FoxO family signaling
Transcription	FOXM1 transcription factor network
	E2F transcription factor network
	RNA Polymerase II transcription termination
	TP53 regulates transcription of cell cycle genes
	TP53 regulates transcription of genes involved in G2 cell cycle arrest
	Gene expression (transcription)
	HIF-1-alpha transcription factor network
Translation	Cap-dependent translation initiation
	Eukaryotic translation initiation
	Translation
	Eukaryotic translation termination
	Eukaryotic translation elongation
	Translation initiation complex formation
	Translation factors

## Data Availability

The data presented in this study are openly available in a GitHub repository at https://github.com/jndeben/phytomeSNPs (28 December 2023).

## Appendix A

**Table A1:** Main PHYTOME study population characteristics, frequencies (n), and means (SDs).

	Total ^(a)^	Group 1 (Standard-Nitrite PHTYOME Meat)	Group 2 (Reduced-Nitrite PHYTOME Meat)
Participants (n)	63	31	32
Females (n)	32	16	16
Males (n)	31	15	16
Age (years)	25.4 (8.5)	25.9 (9.3)	24.6 (7.6)
BMI (kg/m^2^)	22.3 (2.1)	22.0 (2.1)	22.6 (2.1)
Meat intake (g per day)	254 (38)	248 (38)	259 (37)
Physical activity (h per week)	6.5 (3.8)	7.1 (4.2)	5.9 (3.3)

^(a)^ No statistically significant differences between groups 1 and 2.

**Table A2:** Associations between the studied single nucleotide polymorphisms and the risk for colorectal cancer from research in the literature.

SNP	Colorectal Cancer Risk	Study Design	Population	Year	Authors
CYP2E1	Associated with an increased colorectal cancer risk	Case–control	Korea	2019	Kim et al. [64]
	Associated with an increased colorectal cancer risk	Case–control	China	2013	Qian et al. [65]
	Associated with an increased colorectal cancer risk	Case–control	China	2013	Jiang et al. [66]
	Not associated with colorectal adenoma risk	Case–control	USA	2012	Gilsing et al. [67]
	The 96-bp insertion was slightly more frequent in the CRC group	Case–control	Brazil	2012	Silva et al. [68]
GSTM1*0	Associated with a decreased response to a high fruit juice and vegetable diet	Case–control	China	2013	Yuan et al. [69]
	Associated with reduced detoxification of colorectal carcinogens	Case–control	China	2011	Koh et al. [70]
	Associated with an increased colorectal adenoma risk	Case–control	Scotland	2010	Northwood et al. [71]
	Associated with higher micronutrients released from phytochemicals (flavin mononucleotide and 5-MTHF)	Cross-sectional	Greece	2017	Kakkoura et al. [72]
	Associated with decreased DNA repair capacity	Case–control	Slovakia	2012	Dusinska et al. [73]
	Not associated with DNA strand breaks	Crossover	USA	2012	Charron et al. [74]
	Associated with increased antioxidant benefit from Brassica vegetables	Crossover	Italie	2010	Riso et al. [75]
	No statistical interactions were detected between CV intake and GST gene variants on the odds of CRC	Case–control	China	2014	Vogtmann et al. [76]
	High frequency of meat consumption was associated with a four-times increased risk of CRC	Case–control	Poland	2019	Klusek et al. [1]
	Associated with an increased rectal cancer risk	Case–control	India	2011	Wang et al. [77]
GSTT1*0	No significant increase in CRC	Case–control	Poland	2019	Klusek et al. [1]
	Associated with decreased DNA repair capacity	Case–control	Slovakia	2012	Dusinska et al. [73]
	Not associated with DNA strand breaks	Crossover	USA	2012	Charron et al. [74]
	Associated with a decreased response to a high fruit juice and vegetable diet	Case–control	China	2013	Yuan et al. [69]
	Associated with reduced detoxification of colorectal carcinogens	Case–control	China	2011	Koh et al. [70]
	Associated with an increased colon cancer risk	Case–control	China	2011	Wang et al. [77]
	Co-occurrence of GSTM1 and GSTT1 polymorphisms may be an important factor in predisposition to CRC development				
MGMT	Associated with promoter methylation/silencing of MGMT in colorectal cancer	Case–control	USA	2007	Ogino et al. [78]
	Moderate effect on the mutation spectrum in colorectal cancers	Case–control	UK	2005	Halford et al. [79]
	Associated with elevated risk for MGMT-methylated colorectal cancer	Case–control	Australia	2016	Kuroiwa-Trzmielina et al. [80]
	Not associated with colorectal cancer risk	Case–control	USA	2011	Shima et al. [81]
	T allele at SNP rs16906252 is a key determinant in the onset of MGMT methylation in colorectal cancer	Case–control	USA	2009	Hawkins et al. [82]
NAT1*10	Associated with an increased colorectal cancer risk and interaction with meat consumption to modify the disease risk	Case–control	Taiwan	2018	Kamiza et al. [83]
	Associated with increased colorectal adenoma risk	Case–control	USA	2012	Gilsing et al. [67]
	Associated with increased cancer risk	Case–control	Canada	2015	Ho et al. [84]
	Associated with increased colorectal cancer risk	Case–control	China	2012	Liu et al. [85]
	No association with colorectal cancer risk	Case–control	China	2012	Cai et al. [86]
	Red meat consumption significantly increased colorectal cancer risk for NAT1*10 carriers	Case–control	Germany	2006	Lilla et al. [87]
NAT2*7	No interaction between NAT2 genotype and red meat intake in mediating risk of colorectal cancer	Case–control	USA	2015	Ananthakrishnan et al. [88]
	Associated with increased colorectal cancer risk	Case–control	Brazil	2011	da Silva et al. [89]
	Associated with modifying the association between red meat consumption and colorectal cancer risk	Case–control	USA	2015	Wang et al. [90]
	Associated with increased colorectal cancer incidence	Case–control	Jordan	2012	Mahasneh et al. [91]
	Associated with increased colorectal cancer risk	Case–control	China	2012	Liu et al. [85]
NQO1*2	Associated with an increased colorectal cancer risk	Case–control	China	2013	Peng et al. [92]
	Associated with an increased colorectal cancer risk	Case–control	Caucasian population	2012	Ding et al. [93]
	Associated with an increased gastric cancer risk	Case–control	Caucasian population	2013	Lajin and Alachkar [94]
